# Responding to COVID-19: Lessons Learned from a Senior Living and Social Service Organization

**DOI:** 10.3390/geriatrics5040098

**Published:** 2020-11-26

**Authors:** Margaret Danilovich, Christie Norrick, Rachel Lessem, Laura Milstein, Nikki Briggs, Rebecca Berman

**Affiliations:** 1 CJE SeniorLife—Leonard Schanfield Research Institute Chicago, Chicago, IL 60645, USA; christie.norrick@cje.net (C.N.); Rachel.lessem@cje.net (R.L.); milst035@umn.edu (L.M.); nikki.briggs@cje.net (N.B.); Rebecca.berman@cje.net (R.B.); 2 Department of Physical Therapy and Human Movement Sciences, Feinberg School of Medicine, Northwestern University, Chicago, IL 60611, USA

**Keywords:** COVID-19, older adults, retirement communities, crisis response

## Abstract

This case study analyzes one senior living and social service organization’s coronavirus (COVID-19) crisis response. We conducted interviews with *n* = 14 department managers to explore the organization’s pivot to remote work and service provision. We used the Pearson and Mitroff Crisis Management Framework to organize themes. A pre-existing culture of teamwork, willingness to adapt and adopt new approaches, and responsiveness to new policies and procedures facilitated the COVID-19 crisis response. However, low levels of digital literacy among staff, decreased job satisfaction due to no face-to-face interaction between care recipient and service provider, and lack of proactive policies for crisis response, which decreased the speed of enacting remote service provision, were obstacles in effective crisis response. Lessons learned from this case study highlight the need for pre-emptive policy creation on remote service provision and work from home policies, as well as training considerations for senior living and social service organizations.

## 1. Introduction

Older adults and the social service agencies or senior living communities that provide their care have acutely felt the impact of the coronavirus (COVID-19) pandemic. Older people are at a higher risk for COVID-19-related hospitalization, intensive care admission, and mortality [1]. To date, eight out of ten COVID-19 deaths in the US have been adults age 65 and over [2]. Furthermore, the medical syndrome of frailty [3] and many of the pre-existing conditions prevalent among older adults, such as high blood pressure, diabetes, and chronic lung disease, increase mortality risk with a COVID-19 diagnosis [2]. The risk of social isolation increases with age [4] and social isolation and loneliness are associated with increased health risks [5]. Pandemic-imposed isolation may disproportionally affect older adults, especially those with already limited social connections.

In the United States, the state of Illinois put in place strict shelter-in-place policies in response to the increased risk for older adults in contracting COVID-19. On March 11th, 2020, all individuals entering residential senior living building and nursing homes were required to complete COVID-19 screening and temperature checks [6]. By March 17th, all non-essential visits were restricted and group activities and congregate dining were cancelled to decrease disease spread in these congregant living environments. For all persons in Illinois, a shelter in place order issued on March 20th restricted travel to only essential locations, closed non-essential businesses, and forced senior living and social service organizations to rapidly pivot to remote work and cease activities such as in-person socialization groups, which are often a hallmark of their services.

Historically, most services in the senior living industry and in the provision of social services are face-to-face, given the nature of the services, the lack of insurance reimbursement for telehealth services [7], the communication needs of older adults that are often more easily facilitated in person, and the lack of infrastructure and capital funds, particular in many not-for-profit social service organizations, for telehealth solutions. While there have been initiatives to create or increase the uptake of online technology in the field of aging, these innovations have not rapidly been adopted both in in-home care and senior living facilities [8]. Furthermore, less than half of people age 80 and older use the internet on a daily basis, and for those with incomes under $30,000, only 27% routinely access the internet [9]. The combination of these individual and organizational factors makes online or other telehealth service access and provision challenging in the senior living and social service industries.

In response to the COVID-19 pandemic, social service organizations and senior living providers were challenged to rapidly pivot to provide needed services in a remote (e.g., not face-to-face) fashion and engage increasingly isolated older adults populations in creative ways. Given the health and safety crises that arose as a result of the COVID-19 pandemic, the purpose of this paper is to analyze CJE SeniorLife’s crisis management response to provide a case study of an organizational response in the senior living and social service industry in response to the pandemic to share lessons learned for other providers in this space.

## 2. Materials and Methods

### 2.1. Study Setting

Established in 1971, the mission of CJE SeniorLife is to enhance quality of life and facilitate independence of older adults by offering residential services (low-income subsidized housing and assisted living), healthcare services (nursing home care and mental health counseling), and community services and programs (adult day services, care management, educational programs, home delivered meals, and support groups). Serving nearly 20,000 older adults and family members annually, approximately 67% of clients have incomes below 200% of the federal poverty line. There is also a large immigrant population with 53% of clients speaking a language other than English.

### 2.2. Study Design and Analysis

For this case study, we conducted semi-structured individual interviews with department managers to explore how their teams pivoted to remote work and service provision. Since these interviews were conducted under the agency’s continuous quality improvement framework, an ethics review was not conducted. Participants verbally consented to the interview process, including audio-recording. Interview guide questions centered on the experience of working from home, as well as the experience of providing older adult services and programs virtually. Interviews lasted between 30 and 45 min, were audio-recorded, transcribed, coded, and analyzed by two authors (MD and CN) using Microsoft Excel version 14.3.0 following a deductive approach [10] to identify overall themes. Data were kept secured on password-protected files on password-protected computers. Integrity of results were ensured by having each author code transcripts for themes and discuss any discrepancies. As the goal of this project was to understand the organization’s response to the pandemic crisis, interview guide questions were derived around the Input–Process–Output model [11] to identify inputs, processes, and outputs of the crisis response. Inputs are circumstances that exist prior to an event, processes are the interactions between organization members, and outputs are the results of the organization’s response. Then, we used the Pearson and Mitroff Crisis Management Framework [12] to analyze the themes that emerged from manager perspectives of the agency’s response to the COVID-19 pandemic.

### 2.3. Crisis Management Framework

The Crisis Management Framework [12] evaluates interdependency among four key crisis variables: crisis types, time phases, organizational systems, and critical stakeholders to provide a multi-faceted analysis of an organization’s crisis preparedness and response. Details of this model are shown in Table 1. In this case report, we focus specifically on the organizational systems influencing crisis response.

## 3. Results

We conducted 14 semi-structured interviews with departmental managers across the agency from departments such as counseling, care management, IT, nursing home care, affordable housing, and adult day services. Overall, managers viewed the pivot to remote work and providing remote services as a result of the COVID-19 pandemic as feeling “uprooted” and “challenging”. For staff working in the senior living environments, the pandemic experience only added to the strain of pre-existing industry-wide issues of staffing levels and high turnover as staff testing positive for COVID-19 could not work and others with health issues or living in homes with immunocompromised individuals often selected to take Family and Medical Leave Act (FMLA) benefits, leaving teams with increased workload despite the agency maintaining all regulatory requirements for staffing at all times. Furthermore, with the pandemic, there was a dramatic uptake in the requests for counseling and home-delivered meals among new clients, in addition to an increase the amount of services requested by existing clients. The COVID-19 crisis was particularly stressful given that it was both a human/social/organizational crisis, as well as a technical/economic one (Table 1). From a time-phase perspective, early signals of the pandemic came in February and early March as cases were reported in the United States. These early signals served as the impetus for leadership in the organization to purchase supplies such as personal protective equipment (PPE), disinfection materials necessary for the damage containment of the coronavirus, and to prepare for staff to work remotely.

### 3.1. Organizational Systems Influencing Crisis Response

#### 3.1.1. Technical: Materials and Equipment Necessary to Complete Work Tasks

One of the most consistent themes regarding the materials and equipment necessary to respond to the COVID-19 pandemic was the rapid response from the executive leadership team in securing PPE necessary to protect the safety of residents, clients, and staff, as well as equipment to work from home. Prior to the pandemic, the majority of staff had desktop computers in their offices or cubicles, but when the possibility of a shelter-in-place order being issued in Illinois became evident, equipment necessary for working from home such as laptops, tablet computers, scanners, and printers were quickly deployed across the organization. Once the Centers for Medicare and Medicaid Services approved reimbursement for mental health telehealth services, equipment such as tablets and webcam-enabled computers with telehealth software and relevant Health Insurance Portability and Accountability Act (HIPPA) policies were established for counseling staff. However, counselors estimated that nearly 50% of clients did not have home internet or a smart phone, which made video telehealth services impossible to provide, and counselors relied on telephonic visits for these older adults.

In addition, the COVID-19 pandemic significantly disrupted the provision of activity programs throughout both the senior living residences and in community programs. In response, the Community Engagement department rapidly pivoted to use technology such as Zoom and YouTube to deliver programs virtually, which was a new experience, as all of their previous program delivery had been face-to-face. Since CJE SeniorLife did not previously have accounts for the software or use them for the delivery of programming, new policies from the Compliance and Risk Management department had to be quickly instituted, as well as training for staff on strategies for hosting sessions on these platforms. One of the key lessons learned was for two staff members to be on each Zoom program: one leading the program and one serving as the moderator to admit participants to the program and manage any technical issues that participants encountered. Further, we learned it was easier for older adult participants to have one Zoom link for all programs rather than manage different links for various programs. While easier for participants, the disadvantage was the inability to offer concurrent programming.

In the absence of organization guidelines on remote work, managers had to rapidly create new policies and procedures on the use of technology, remote work, supporting staff, and best practices on supervising employees in a remote work environment. One challenge was creating digital or electronic materials for documentation previously done on paper. Furthermore, educating clients on how to reach staff and access services in a remote fashion was challenging, particularly as many clients had limited to no technology infrastructure in their homes. The lack of internet access was particularly challenging in the low-income, subsidized housing buildings where internet is not a standard amenity and residents often lacked funds to purchase internet for themselves. To maintain communication, staff made weekly telephone calls with clients, which required a significant amount of staff time to achieve.

#### 3.1.2. Human: Integration of People and Equipment to Evaluate How Technical Systems Fit with Human Users

The most significant challenge reported by managers was teaching staff how to use technology to support remote work and service provision, primarily due to low levels of technical proficiency and digital literacy. For some staff, this was the first time they had worked on a laptop computer, and unstable home internet was an obstacle in working from home. For departments such as Care Management, which routinely used laptops to serve clients in the field prior to the pandemic, staff experienced little interruption in shifting to remote work. Managers felt the IT department had limited time to conduct staff training on how to use new devices given their increased workload in deploying devices and setting up software packages necessary to work from home, and this responsibility often fell to department managers. One manager created instructions on “how to work from home” including tips on using hotspots, strategies to manage work–life balance, and advice for structuring one’s day when working remotely. Another manager stated a month into the crisis: *“I’m still doing a lot of trouble-shooting with staff members around simple things like how to open up Microsoft.”* Despite the low level of staff proficiency with technology at the beginning of the pandemic, managers reported that staff members were willing to adopt and engage in new approaches to complete their work, such as using fillable PDFs instead of printed paper forms.

As a result of the lack of computer and internet access among many older adult clients, managers had to rely upon subjective reports from clients over the telephone versus seeing the home environment or the physical performance using the video features on telehealth platforms. One manager noted, *“To do an assessment (subjectively) and have a full assessment of ADLs and IADLs to get home care is really hard. Clients don’t really have any technology in order to access any services digitally. And you know…we can’t really trust them. They say they don’t have trouble, but you know they just aren’t telling you.”* As a result, documentation and assessment forms were modified to document subjective client reports versus objective assessments from staff. While this approach gives more voice to the client, managers reported staff felt as if they could not fully trust the client’s report of items such as difficulties with instrumental activities of daily living without actually seeing the client perform these tasks.

Prior to the pandemic, one of the assumptions that some staff had was that clients did not have access to technology nor would they want to participate in online programs. In moving services remotely, the organization had no choice but offer programs through Zoom and pre-recorded videos on YouTube. The various departments enacted such online programs at different time points with some departments being early adopters, while others waited until there was some evidence that older adults would actually participate in these programs. One of the clear advantages we saw was the ability to reach older adults who would not typically attend programs due to living outside of the area. For example, a weekly group fitness class was attended by older adults who lived over an hour away from the main organization headquarters, and these individuals stated that had it not been for Zoom, they would never attend programs at the organization due to the distance. In particular, in the nursing home and assisted living residences, it was rare to offer residents live-streaming and pre-recorded programs in favor of face-to-face activities to promote social engagement. In implementing online programming in the nursing home and assisted living, there was an increase in the number of residents participating in activities compared to the pre-COVID period with a subsequent reduction in staff time to transport residents to and from activities. Programs were offered through an internal TV channel such that all residents could view the program in their own room. An addition benefit from the online programming model was leveraging shared resources across the organization. Rather than art therapists working in-person with residents at the each of the nursing home, assisted living, and adult day services locations, online programming allowed for one art therapist to create a program that could be widely shared more efficiently. A manager felt there were improved efficiencies in staffing by using online options, noting that: *“Creating one program and then replicating it is certainly a lot easier than everybody trying to create a program.”* However, managers noted that the online programs led to more passive engagement of residents in watching an activity versus active engagement in participating in face-to-face activities that provided cognitive and psychomotor stimuli.

#### 3.1.3. Infrastructure: Communication Channels to Manage the Crisis

Communicating regularly with team members was an important strategy used to manage the crisis. With the move to work from home, departments that previously met face to face for weekly staff meetings moved to virtual formats. There was a pre-existing culture of teamwork at CJE SeniorLife, and this culture persisted during the pandemic. One of the key elements to facilitate communication within departments was the introduction of daily huddles, short meetings each morning to review client needs, staffing issues, and updates for the day ahead. One manager noted, *“We now meet (on Zoom) at 9:30 every morning. At work, this was much more informal because we saw each other each day and we only officially met once per week.”* For departments such as Counseling and Care Management, where staff needed time to present patient cases and get clinical supervision input, Zoom meetings allowed staff to connect and see each other, which was viewed by managers as advantageous over telephone conference calls because it allowed staff to see each other’s facial expressions and body language. In the first few weeks of the pandemic crisis, all managers met over Zoom twice-weekly to share information and communicate crisis management plans across departments. As work activities stabilized as the pandemic progressed, these meetings decreased to once per week, but managers continued to view these as positive means to communicate plans for re-opening buildings and services.

Managers reported managing client and resident stress and anxiety during the pandemic to be a challenge. Weekly telephone calls were used across the agency to check in with clients and Zoom visits with family for nursing home and assisted living residents proved effective in maintaining some form of communication channel with older adults. However, this required a tremendous effort in social worker and other staff time to coordinate appointments with families and facilitate the Zoom meeting. In the volunteer department, the manager established a private Facebook group for volunteers so they could connect and stay engaged with each other, while volunteer work was paused due to visitor restrictions in residential buildings. This proved effective to keep volunteer engagement levels high, despite not offering volunteer opportunities.

One of the most important resources to help social worker and care management staff assist clients specifically related to the COVID-19 pandemic was the creation of an internal COVID resource guide. One social worker served as the resource coordinator and compiled resources from organizations across the Chicagoland area such as food and medicine deliveries, financial assistance to access federal and state COVID relief funds, reduced cost internet access, and sources for pro bono legal or advance care planning, and they also updated the guide weekly as new resources became known. Managers reported this resource guide was particularly valuable to centralize and rapidly communicate changing resources for older adults across departments.

#### 3.1.4. Cultural: Safety Practices, Attitudes, and Organizational Orientation that Influenced Crisis Response

As aggressive litigation has become more common in both senior living and nursing home industries, CJE SeniorLife has a strong orientation toward a culture of safety and conservative practices in security and client privacy, which shaped the organization’s crisis response to the pandemic. For instance, in order to shift to remote services and work from home, new policies from the risk management department needed to be written and enacted, especially to comply with the Health Insurance Portability and Accountability Act (HIPAA). One manager noted that getting the approval required to move forward with virtual services from risk management and corporate compliance offices *“felt like it was taking forever”.* This perceived delay contributed to uncertainty among staff on how to perform work tasks remotely and increased strain. However, interviews indicate that staff were responsive to new policies and procedures once created.

The organization also had to redeploy staff from services closed due to COVID-19, such as adult day services, to other departments to prevent staff furlough. Minimizing stress for relocated staff required attention to staff attitudes about teamwork and an organization orientation of adaptability. The organization facilitated successful transitions by trying to keep teams intact in redeployment. For example, the entire adult day service team transitioned to working in the nursing home, including their manager, who felt that staying together promoted cohesion and camaraderie.

For staff who worked remotely, a frequent theme reported among managers was that the lack of face-to-face interaction with clients was demoralizing and the lack of human connection was a strain on staff members’ own mental health. Recognizing this strain, one manager instituted a team-building ritual of explicitly reminding staff to “take a breath” each day and began all meetings with a moment for mindfulness while ending meetings with a statement of gratitude whereby staff each stated one thing for which they were grateful. Conversely, some managers in non-client facing roles found work from home made them more productive, but they still missed the social aspects of work life. A manager reported: *“We’re able to be more focused at home and don’t have as many interruptions... but I’m missing other people to ‘vent’ with and talk to. I’m not taking an hour for lunch... I’m working constantly.”*

A number of staff members were very concerned about contracting COVID-19 either in the course of their direct work with clients or on public transportation commuting to work. The organization provided staff with the option to take paid or unpaid time off while still maintaining their position to support employees who were older, immunocompromised, or had members of their household who were immunocompromised. Further clients were highly fearful. One manager reported, *“They don’t want to touch the mailbox, they are scared to go to the grocery store,”* and this fear, coupled with the lack of digital access made already isolated older adults even more isolated and challenging to provide services for.

#### 3.1.5. Emotional/Belief: Attitudes and Beliefs, Particularly of Senior Leadership, on the Organization’s Crisis Response

At the staff level, attitudes and beliefs prioritizing serving clients directed the crisis response. However, this dedication also had downsides in terms of contributing to staff burn out. One manager voiced concern that, *“My staff [are] all part-time and it is a challenge to limit them to only working their hours. Staff [are] working outside of their scheduled hours but only [are] getting paid for the hours they normally would do. It is hard to stop working when you are at home. Because they are running daytime programs, they are responding to emails at night and never signing off.”*

The primary focus of senior leadership was on securing PPE and other necessary items to keep residents and staff safe. The CEO used a mass-messaging service to provide frequent updates to staff on the number of COVID-19 cases in the nursing home and assisted living, as well as return to work plans. In addition, “town halls” on Zoom provided an opportunity for staff to have specific questions addressed. Further, the leadership provided a hazard pay bonus for front-line staff working with patients who tested positive for COVID-19 in the nursing home or assisted living to help support better staffing ratios and demonstrate appreciation for staff in these roles. This focus was appreciated by managers who noted that this priority was *“very helpful in knowing they (senior leadership) are supporting everything we’re doing”*. However, other managers noted that HR initiatives previously implemented in the face-to-face work environment such as monthly birthday parties, staff appreciation days, and employee recognition efforts stopped in the face of the pandemic, which led to a deterioration of morale in the face of an already existing stressful work environment. A manager stated, *“We need to figure out how to remotely reward people and recreate the energy from the office into work from home. We need to emotionally support people and find more ways to acknowledge people in a broader sense to publicly acknowledge little successes in the team to build morale.”*

## 4. Discussion

The purpose of this case report was to analyze one older adult senior living and social service organization’s response to the COVID-19 pandemic to share lessons learned and insights into how an organization pivoted and responded to the crisis. Discussion points are summarized in Table 2.

Given the traditional methods by which senior living and social service agencies tend to operate (e.g., heavy emphasis on in-person service provision and slow adoption of technology [13]), investigating the response following a crisis event that dramatically disrupts operations can provide important lessons for others in the industry. The results of manager interviews provide concrete examples of organizational learning using the Crisis Management Framework. The critical components that managers reported facilitated a positive crisis response included a culture of teamwork, willingness to adapt and adopt new approaches, and responsiveness to determine new policies and procedures. The biggest challenges faced in responding to the crisis included low levels of digital literacy among staff, the lack of interaction between care recipient and service provider decreasing job satisfaction, and the lack of centralized policies and procedures for crisis response which strained managers further in creating new materials.

One of the key ways to navigate a crisis is to prepare for a crisis before it begins. While there were signs that COVID-19 was spreading and could become a pandemic, the most important leadership actions and inputs for the crisis response was in securing PPE and opening communication channels around pivoting to remote services and work from home. Despite these early efforts, managers consistently reported that the transition was challenging, particularly in an industry so entrenched in face-to-face services. To prepare for future crises and industry shifts in light of increasing online services, senior living communities and social service agencies should prepare contingency plans and remote work procedures to ensure the technical, human, and infrastructure systems necessary for care provision. Further, we found managers were strained in knowing how to supervise staff remotely and transition services to digital platforms. This finding suggests a need for human resource departments to take an active role in disseminating organizational management best-practices for virtual supervision of staff, as well as the role of IT departments to provide training and educational programs to overcome digital literacy hurdles. Others have noted the knowledge gaps in social work education and practice in the areas of digital literacy and technological competency [14]. Our findings contribute to this emerging body of knowledge and create a call for action for those in the senior living and social service organization industries to ensure adequate digital competencies of staff before shifting to telehealth or other virtual care models.

Our finding that the lack of face-to-face interaction was perceived to reduce job enjoyment is of note given that even before COVID-19, there has been movement toward increasing telehealth options among older adult service providers. Other research has shown that staff perceive video telehealth services to provide peace of mind and a strong sense of job satisfaction [15]. In this case study, it is possible that the low rates of technology access among our low-income older adult population did not provide as many opportunities for video connection with clients, which could explain why managers and staff reported lower morale with client interactions. As remote services and telehealth become more ubiquitous, consideration should be placed on how to best replicate the satisfaction and joy of helping older adults in face-to-face care.

As shelter in place restrictions have eliminated in-person and group activities, it has become harder for older people, especially those who live alone, to remain socially engaged. While technology is a powerful solution to promote engagement while social distancing, there remain many barriers to technology use and access among older adults including lack of instruction, knowledge, cost, particularly those who are in retirement living or skilled nursing communities [16]. Importantly, we found that these technology barriers are not only on the part of the older adult clients but for staff as well. The lower digital proficiency levels among staff were enormous hurdles to remote working and service provision. To prepare staff for delivering more telehealth and remote services, senior living and social service agencies will be challenged to adequately prepare this workforce through targeted education and training to build confidence in their technology skills.

There are several notable limitations of this case report. First, this report summarizes the interview results of one senior living, social service organization only, and the results may not be generalizable. Second, participants completing interviews with the authors may not have been completely transparent in describing their experiences during the COVID-19 pandemic, making the conclusions drawn less reliable. Lastly, because the authors of this case study are also employees of CJE SeniorLife, it is possible that their own impressions and feelings about the organization’s COVID response colored their interpretation of the data. While the authors did not formally complete any member checking with stakeholders, the authors’ presence within the organization provided a type of observation and triangulation to ensure credibility of interview reports and themes. Despite these limitations, this case report offers valuable information about the crisis management strategies employed by a senior living and social service agency to others in the industry.

## 5. Conclusions

The strategies for enhancing the effectiveness of remote work and services in this case example can be applied in other organizations that provide senior living and social services. To prepare for future crises, senior living communities and social service organizations will need to prepare contingency plans and remote work procedures. While social service providers are non-medical providers, they may wish to borrow team communication techniques from the medical setting. While rigorous research of huddles is in its infancy [17], the early accounts of this team communication technique report favorable success in improving knowledge transfer and efficiency. This technique may be of even greater value outside of the face-to-face environment to ensure teamwork and facilitate safety and client outcome achievement. In conclusion, proactive policies and procedures for facilitating work from home, including targeted educational sessions to improve digital literacy, are likely needed to help those in the senior living industry and social service agencies more effectively provide remote care and services.

## Figures and Tables

**Table 1:** Pearson and Mitroff Crisis Management Framework Model.

Crisis Variable	Components and Description
Crisis Type	Technical or economic crisis Human, social, or organizational crisis
Time Phases	Period of time in the crisis: Early signals Prevention Damage containment Recovery Organizational learning
Organizational Systems	Technical: materials and equipment necessary to complete work tasks Human: integration of people and equipment to evaluate how technical systems fit with human users Infrastructure: communication channels and crisis management teams with clearly defined roles and responsibilities Culture: safety practices, attitudes, and organizational orientation that influence the physical, financial, and human resources response to a crisis
Critical Stakeholders	All parties who may make a crisis easier or more difficult to manage

**Table 2:** Staff Quotes of How the Organizational System Was Impacted by the COVID-19 Crisis.

Organizational System Crisis Variable Components and Description	Supporting Quotes Provided by Managers Showing How the Component Was Addressed or Impacted in the COVID-19 Crisis
1. Technical: materials and equipment necessary to complete work tasks	1. *“Our private Facebook group has been a great way to connect with volunteers remotely”*
2. Human: integration of people and equipment to evaluate how technical systems fit with human users	2. *“There is a great need to make volunteers and staff more tech savvy”*
3. Infrastructure: communication channels and crisis management teams with clearly defined roles and responsibilities	3. *“We don’t do any electronic documentation. I* (as manager) *needed to figure out a way for us to do documentation so I transitioned us to fillable PDFs”*
4. Culture: safety practices, attitudes, and organizational orientation that influence the physical, financial, and human resources response to a crisis	4. *“*(Work from home) *is challenging because of the lack of socialization…we are on top of each other in our space, so we have natural collisions. It’s challenging to create that in a structure way remotely.”*
5. Emotional/beliefs: attitudes and beliefs, particularly of senior leadership, on the organization’s crisis response	5. *“We need training on technology and webinars on how to not only take care of clients, but also how to take care of yourself”*
